# Clinical Characteristics and Prognosis of Gastrointestinal Metastases in Solid Tumor Patients: A Retrospective Study and Review of Literatures

**DOI:** 10.1155/2019/4508756

**Published:** 2019-12-20

**Authors:** Li Lin, Xiangyi Wang, Chuanhao Tang, Jun Liang

**Affiliations:** ^1^Department of Oncology, Peking University International Hospital, Life Park of Zhongguancun, Changping District, Beijing, China 102206; ^2^Department of Oncology, Beijing Ditan Hospital, Capital Medical University, Jingshun East Road, Chaoyang District, Beijing, China 100015

## Abstract

**Background:**

According to the literature and our experience, patients with gastrointestinal metastases are relatively rare. Numerous case reports and literature reviews have been reported. We present one of the larger case series of gastrointestinal metastases.

**Objectives:**

To explore the clinical characteristics and prognosis of patients with gastrointestinal tract metastases, which are rare metastatic sites.

**Methods:**

Patients with gastrointestinal metastases in the setting of stage IV primary carcinomas treated at Beijing Ditan Hospital and Peking University International Hospital from November 1992 to August 2017 were included in this study. The diagnosis of gastrointestinal tract metastases was based on histopathology.

**Results:**

30 patients (median age 56 years, 56.7% female) were included. The most common primary carcinomas associated with gastrointestinal metastases were breast (11 patients, 36.7%), stomach (9 patients, 30.0%), and lung (4 patients, 13.3%) cancer. The major pathological types were adenocarcinoma (16 patients, 53.3%) and ductal carcinoma (9 patients, 30.0%). Ten patients (33.3%) underwent local gastrointestinal treatment, and 20 patients (66.7%) underwent nonlocal treatment (involving chemotherapy alone or best supportive care). For breast cancer patients and gastric cancer patients who underwent local therapy, a significant survival advantage was observed (*p* = 0.001 and *p* = 0.012, respectively). The presence of other common metastases was identified as an independent poor prognostic factor through multivariate analysis with a HR (hazard ratio) of survival of 0.182 (95% confidence interval (CI) 0.11-0.523, *p* = 0.031).

**Conclusion:**

Gastrointestinal metastases are most frequently from breast invasive ductal carcinoma. The presentation of other common metastases with gastrointestinal metastasis indicates poor prognosis, and selected patients may benefit from surgical intervention.

## 1. Introduction

Gastrointestinal metastasis refers to the growth of malignant cells from the primary tumor site through the blood vessels, lymphatic vessels, or other pathways to the stomach or intestine. Clinically, gastrointestinal metastases are relatively rare. The most common primary tumor associated with gastrointestinal metastasis is breast cancer [1, 2], with a frequency of 8-12%. Due to its extremely low incidence, it is easily misdiagnosed as a primary gastrointestinal tumor [3–5], which affects the clinical therapy regimen, shortens the survival period, and affects the patient's quality of life and prognosis.

Due to the small number of cases of metastatic gastrointestinal tract carcinoma, the current literature mostly consists of case studies, and it is impossible to effectively summarize the characteristics of gastrointestinal metastases. In order to further understand the clinical characteristics of this disease, this review analyzed the clinical data of 30 cases of metastatic gastrointestinal cancer in our hospitals to explore the clinical characteristics, originating primary carcinoma, and prognosis.

## 2. Patients and Methods

Patients enrolled in this study were diagnosed with gastrointestinal metastases at the onset or progression of primary tumor at Beijing Ditan Hospital and Peking University International Hospital from November 1992 to August 2017. All patients signed the informed consent for participation. The diagnosis of gastrointestinal metastases was based on histopathology. Metastases located in the muscle/subcutaneous tissues were based on histopathology and clinical manifestations. The other metastatic sites (e.g., brain, kidneys, pancreas, or spleen) were diagnosed by computed tomography (CT), magnetic resonance imaging (MRI), and positron emission tomography/computed tomography (PET/CT) or histopathology.

Data were collected from the clinical medical records, including name, gender, age, time of diagnosis, pathological type, and distant metastasis. All patients were followed up, including telephone follow-up or review of medical records, to determine the patient's survival and to calculate survival time (accurate to the month). Survival was defined as the time from detection of gastrointestinal metastases until date of mortality or final follow-up. Median survival and overall survival were calculated.

Univariate analyses were performed using the Kaplan-Meier method, and groups were compared using a log-rank test. Multivariate analysis used the Cox proportional hazards model. All analyses were performed using the SPSS 21.0 software program (IBM SPSS, Armonk, NY, USA). All statistical tests were two-sided, and *p* < 0.05 was deemed to indicate statistical significance.

## 3. Results

Totally, 30 cases of metastatic gastrointestinal carcinoma were included. The clinicopathological characteristics of patients with gastrointestinal metastases are presented in Table 1. The median age at the time of detection of gastrointestinal metastases was 56 years (range, 34-79 years; mean, 57.3 years). Thirteen patients were male (43.3%) and 17 patients were female (56.7%).

**Figure 1:**
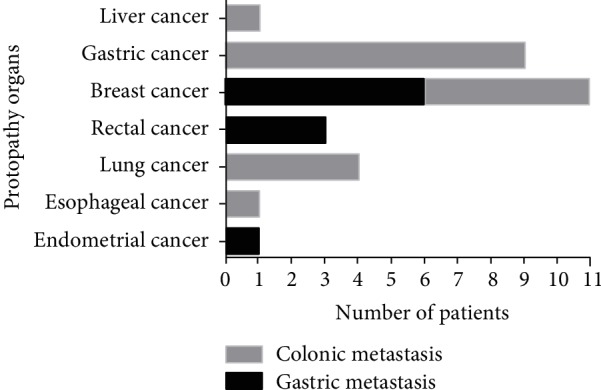
The frequency of protopathy.

Nine patients (30%) were diagnosed with metastatic gastric carcinoma. Their primary cancer sites, in decreasing order of frequency, were breast cancer (5 patients, 55.6%), rectal cancer (3 patients, 33.3%), and endometrial cancer (1 patient, 11.1%). Four patients (44.4%) were identified with a history of breast invasive ductal carcinoma, one patient (11.1%) had breast lobular carcinoma, and four patients (44.4%) had adenocarcinoma. Twenty-one patients were diagnosed with metastatic colonic carcinoma, and the primary cancer sites, in decreasing order of frequency, were gastric cancer (9 patients, 42.9%), breast cancer (6 patients, 28.6%), lung cancer (4 patients, 19.0%), liver cancer (1 patient, 4.8%), and esophageal cancer (1 patient, 4.8%). The pathological types included adenocarcinoma (6 patients, 28.6%), signet ring cell carcinoma (1 patient, 4.8%), breast invasive ductal carcinoma (6 patients, 28.6%), hepatocellular carcinoma (1 patient, 4.8%), squamous carcinoma (1 patient, 4.8%), and sarcoma (1 patient, 4.8%) (Figure 1).

Synchronous metastases were defined as pathologically confirmed gastrointestinal metastases identified at the time of primary cancer diagnosis or within one month. Metachronous metastases were defined as gastrointestinal metastases diagnosed more than one month after initial diagnosis of the primary cancer. Fourteen patients (46.7%) were found to have synchronous metastases, and 16 patients (53.3%) had metachronous metastases. The median interval from the time of primary cancer diagnoses to metastatic presentation was 22.92 months (range, 0-209.94 months; mean 42.82 months).

The most common metastatic sites, in decreasing order of frequency, were bone, distant lymph nodes, the lung, the liver, adrenal glands, the ovary, the brain, the peritoneum, and the pelvic cavity. Less common metastatic sites, in decreasing order of frequency, were soft tissue, bone marrow, the breast, and the spleen (Table 1). Five patients sought initial consultation for gastrointestinal metastatic symptoms prior to metastases to other sites.

Of the 30 patients described in our study, 23 patients received systemic treatment and the other 7 patients only received best supportive care due to poor physical score or poor financial condition. Detailed treatment regimens for all patients are shown in Table 1. After diagnosis of gastrointestinal metastases, 10 patients received surgical intervention, which included gastrointestinal palliative surgery, radiation therapy (including particle implantation), and radiofrequency ablation.

## 4. Survival Analysis

**Figure 2:**
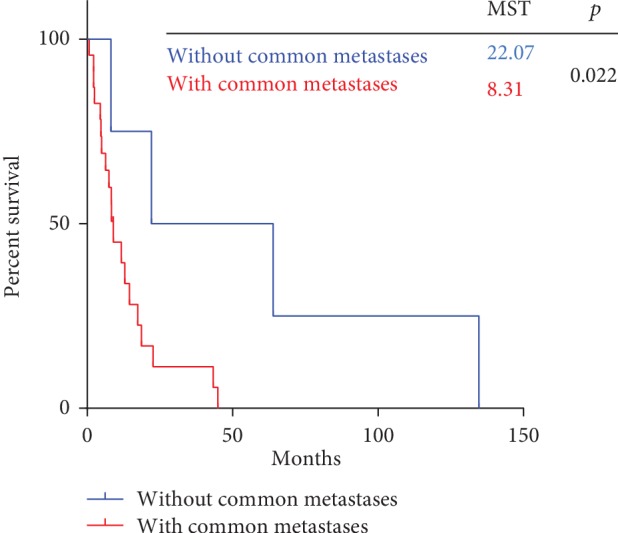
Survival after diagnosis of gastric or colonic metastasis.

**Figure 3:**
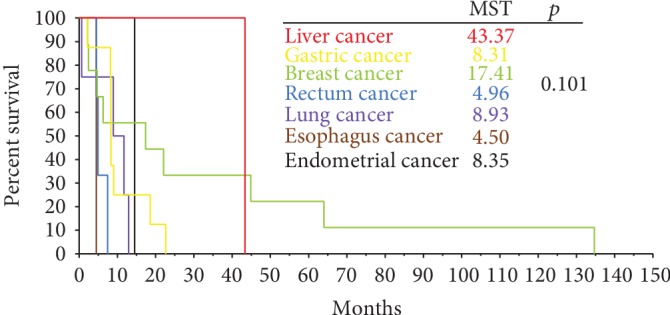
Survival after diagnosis of common metastases.

Follow-up data were available for 30 patients. The median survival was 8.35 months (95% CI 7.18-9.52 months). As shown in Figure 2, the median overall survival (mOS) after diagnosis of gastrointestinal metastases was significantly shorter in patients with metastases to common sites than in those without common metastases (mOS 8.31 months [95% CI 6.87-9.75 months] vs. 22.10 months [95% CI 0.00-9.75 months], *p* = 0.022).  Figure 3 shows the mOS by primary cancer type (*p* = 0.101).

**Figure 4:**
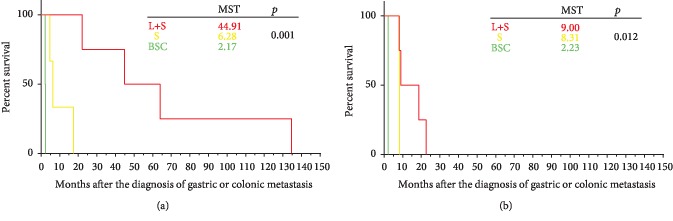
(a) Survival of breast cancer patients after diagnosis of gastric or colonic metastasis, based on treatment. (b) Survival of patients with gastric cancer after diagnosis of colonic metastasis, based on treatment. S: systemic treatment; L: local treatment; BSC: best supportive care.

There was no significant difference in mOS among the 30 patients based on the treatment regimen. However, as shown in Figure 4(a), among the 11 breast cancer patients, mOS was significantly longer among patients who received systemic therapy plus local treatment compared to those who received systemic therapy alone and those who received best supportive care (mOS 44.91 months [95% CI 3.86-85.96 months] vs. 6.28 months [95% CI 3.75-8.80 months] vs. 2.17 months, *p* = 0.001). According to the Kaplan-Meier analysis, as shown in Figure 4(b), systemic therapy plus local treatment was superior to systemic therapy as well as best supportive care (mOS 9.00 months [95% CI 0.00-19.20 months] vs. 8.31 months [95% CI 8.21-8.42 months] vs. 2.23 months, *p* = 0.012).

Metastases to common sites were found to be independent poor prognostic factors through a multivariate stepwise Cox regression analysis. Common metastases were associated with a hazard ratio of 0.18 (95% CI 0.039-0.855, *p* = 0.031).

## 5. Discussion

So far, we have not got a thorough understanding about metastatic gastrointestinal carcinoma, which was viewed as uncommon metastases. Therefore, we reviewed the clinical information of patients with gastrointestinal metastases at our institution from 1992 to 2017 and analyzed the patients' characteristics and prognosis. As far as we know, it is one of the largest sample sizes of study about metastatic gastrointestinal carcinoma.

We can see from Table 1 that most patients in our cohort were female, due to the large number of patients with breast cancer as a primary tumor. The median time from diagnosis of primary cancer to detection of gastrointestinal metastases was 15.03 months (range 0-197.19 months; mean 34.60 months). Metachronous gastrointestinal metastases were the most common presentation. Metastatic gastrointestinal carcinoma was more likely to be diagnosed later in the disease course. This may be because organs that are uncommon sites for metastasis do not provide a suitable microenvironment to support tumor cell survival and it may take time for tumor cells to evolve to adapt to the hostile microenvironment [6].

In this study, breast cancer was the most frequent malignancy to present with gastrointestinal metastasis, as has been noted in other reports [7, 8]. The most frequent sites of breast cancer metastasis include local metastasis and the distant lymph nodes, brain, lungs, liver, and bones. Ciulla et al. [9] found that 6% of women with breast cancer developed gastric metastasis during the course of the disease. The incidence of gastrointestinal tract metastases observed in autopsy studies varies from 4% to 18% [9, 10], with the most commonly affected organ being the stomach, followed by the colon and rectum. Metastatic dissemination to the upper gastrointestinal tract is related mainly to lobular infiltrative breast carcinoma [11, 12]. In our review, more than half of patients had ductal carcinoma. Five of 8 patients had the lobular subtype in the Pectasides series [13]. In a series published by Taal et al. [14], 83% of the 51 breast patients with gastric metastasis and 88% of the 17 breast cancer patients with colorectal metastases had a lobular subtype as the primary tumor. As is well known, the majority of breast tumors arise in the ductal epithelium. Since the ductal subtype is the most common breast neoplasm, the proportion observed in our series seems reasonable.

The most common sites of gastric cancer metastasis are the liver, peritoneum, lungs, and bones [15]. In the existing literature, only a few case reports [16, 17] and a retrospective study [18] describe a diagnosis of colorectal metastasis from gastric cancer. The retrospective study [18] analyzed 23 gastric cancer patients with intestinal metastasis, of whom only 11 were pathologically diagnosed as having colonic metastasis. Most of the patients had poorly differentiated adenocarcinoma or the signet ring cell type with a propensity to develop into rare intestinal metastasis. Notably, 8 out of 9 patients in our study were diagnosed with poorly differentiated adenocarcinoma, and the other patient had a signet ring cell type.

Primary lung cancer frequently metastasizes to the brain, liver, adrenal glands, and bones. It was reported that non-small-cell lung cancer with uncommon metastases such as the soft tissue, kidney, pancreas, spleen, peritoneum, intestine, bone marrow, eye, ovary, thyroid, heart, breast, tonsil, and nasal cavity tends to indicate a poor outcome [19]. However, the clinical incidence of lung cancer metastasis to gastrointestinal sites has been reported to be as low as 0.2%-1.7% [20–22]. Every type of lung cancer can result in gastrointestinal metastasis. Previous studies [23, 24] reported that squamous cell carcinoma and small-cell lung cancer cause gastrointestinal metastases more frequently than other lung tumor cell types. However, in our study, adenocarcinoma was the most frequent type resulting in gastrointestinal metastases. Consistent with our observations, some clinical studies [22, 25, 26] have shown that adenocarcinoma and squamous cell carcinoma were the most common histological types causing gastrointestinal metastases.

Previous studies reported that the small bowel is the most common gastrointestinal metastatic site for lung cancer [24, 27]. In our study, 1 out of the 4 patients with lung cancer presented with small bowel involvement. Gastric metastases arising from lung cancer are extremely rare. In our study, all 4 lung cancer patients also had colorectal metastases. Only three patients in our study had rectal cancer, one had hepatic cancer, one had endometrial cancer, and one had esophageal cancer. Therefore, more thorough scientific documentation is required to discuss the relevant characteristics of these primary sites.

Although rare, gastrointestinal tract metastases can have a negative impact on survival and must be addressed from the time they are first identified until the date of patient mortality or final follow-up. Because the presence of gastrointestinal metastasis is likely to be a preterminal event [28], the prognosis is poor and the survival period is extremely short. In our cohort, the median survival after diagnosis of gastric metastasis was 8.53 months. Some previous studies [14, 28, 29] advocated systemic therapy for breast cancer with metastasis to the stomach, rather than using surgery as a primary treatment option. This is partly because hormone receptor expression is typically positive in patients with gastric metastases [12, 30]. We hypothesize that the biological features of primary breast tumors may partially contribute to the sensitivity of gastric metastases to hormonal therapy. However, surgery may only be considered in cases of acute complications, including gastrointestinal bleeding, obstruction, and perforation, to improve quality of life.

Other studies [31, 32] also hypothesized that surgery may be the optimal first-line treatment for operable solitary breast cancer metastasis to the stomach. As presented in Figures 4(a) and 4(b), survival analysis of our patient cohort showed that combination therapy (surgical intervention plus systemic treatment) did significantly extend OS of breast cancer and gastric cancer patients (*p* = 0.001 and *p* = 0.012, respectively). In this context, treatment should be individualized and should comprise local and systemic therapy [31], depending on the progression of the disease. The present study indicates that combination treatment strategies, including gastrointestinal palliative surgery, radiation therapy (including particle implantation), and radiofrequency ablation, may be the optimal choice for gastrointestinal metastases from breast cancer and gastric cancer.

Whether or not gastrointestinal metastases are associated with other common sites of metastasis is rarely included in prognostic analyses. We speculated that patients with common metastases would be more likely to have poor outcomes. Notably, gastrointestinal metastases frequently occur synchronously with other sites of metastases. In the current study, systemic metastases to other locations were detected in 25 patients (83.3%) prior to the diagnosis of gastrointestinal metastases and typically occurred in the bone (50%), lymph node (30%), lung (23.3%), and liver (20.0%). We recommend that physicians keep in mind the possibility of gastrointestinal metastasis in patients with these common metastases. In addition, gastroscopy [33] and colonoscopy are strongly recommended to detect gastrointestinal metastasis in patients who present with dyspepsia and other gastrointestinal symptoms or whose tumor marker levels remain elevated after chemotherapy or surgical treatment.

A major limitation of our study is the small sample size. Regardless, it is one of the largest case series of gastrointestinal metastases derived from all carcinoma types to date and may aid clinicians in their treatment efforts. A second limitation of our study is its retrospective design, which spanned a long period of time. The current study focuses on factors contributing to survival and appropriate treatments for patients with gastrointestinal metastases. Prospective studies with a longer follow-up time and larger patient numbers may allow an improved understanding of the biological, pathological, and clinicopathological characteristics, clinical outcomes, and endoscopic features associated with gastrointestinal metastases.

## 6. Conclusions

In conclusion, breast invasive ductal carcinoma is the most common primary tumor associated with gastrointestinal metastases. Cooccurrence with other common sites of metastasis tends to indicate a poor outcome, and selected patients may benefit from tailored treatment strategies.

## Figures and Tables

**Table 1:** Clinical characteristics of patients with gastrointestinal metastases.

	Metastatic gastric cancer		Metastatic colorectal cancer		All	
	*N*	%	*N*	%	*N*	%
Age (years), median (range)	62 (50-79)		55 (34-73)		56 (34-79)	
Gender						
Male	2	22.2	11	52.4	13	43.3
Female	7	77.8	10	47.6	17	56.7
Metastatic time						
Synchronous metastases	4	44.4	10	47.6	14	46.7
Metachronous metastases	5	55.6	11	52.4	16	53.3
Primary carcinoma sites						
Hepatic cancer	0		1	4.8	1	3.3
Gastric cancer	—		9	42.9	9	30.0
Breast cancer	5	55.6	6	28.6	11	36.7
Colorectal cancer	3	33.3	—		3	10.0
Lung cancer	0		4	19.0	4	13.3
Esophageal cancer	0		1	4.8	1	3.3
Endometrial cancer	1	11.1	0		1	3.3
Histology						
Adenocarcinoma	4	44.4	12	57.1	16	53.3
Squamous carcinoma	0		1	4.8	1	3.3
Invasive ductal carcinoma	4	44.4	5	23.8	9	30.0
Invasive lobular carcinoma	1	11.1	0		1	3.3
Sarcoma	0		1	4.8	1	3.3
Hepatocellular carcinoma	0		1	4.8	1	3.3
With common metastasis						
Lymph node	1	11.1	8	38.1	9	30.0
Lung	2	22.2	5	23.8	7	23.3
Liver	1	11.1	5	23.8	6	20.0
Bone	5	55.6	10	47.6	15	50.0
Adrenal gland	0		4	19.0	4	13.3
Ovary	0		3	14.3	3	10.0
Brain	0		3	14.3	3	10.0
Peritoneum	1	11.1	2	9.5	3	10.0
Pelvic cavity	0		3	14.3	3	10.0
With other uncommon metastases						
Bone marrow	1	11.1	2	9.5	1	3.3
Soft tissue	1	11.1	3	14.3	4	13.3
Breast	0		1	4.8	1	3.3
Spleen	0		1	4.8	1	3.3
Treatment						
Gastrointestinal topical therapy (metastasectomy/radiotherapy/radiofrequency ablation/microwave coagulation)+palliative chemotherapy	0		10	47.6	10	33.3
Chemotherapy alone	7	77.8	6	28.6	13	43.3
Best supportive care only	2	22.2	5	23.8	7	23.3

## Data Availability

The data used to support the findings of this study are included within the article.

## References

[1] Taal B. G., den Hartog Jager F. C. A., Steinmetz R., Peterse H. (1992). The spectrum of gastrointestinal metastases of breast carcinoma: I. Stomach. *Gastrointestinal Endoscopy*.

[2] Taal B. G., den Hartog Jager F. C. A., Steinmetz R., Peterse H. (1992). The spectrum of gastrointestinal metastases of breast carcinoma: II. The colon and rectum. *Gastrointestinal Endoscopy*.

[3] Abid A., Moffa C., Monga D. K. (2013). Breast cancer metastasis to the GI tract may mimic primary gastric cancer. *Journal of Clinical Oncology*.

[4] Kim M. J., Hong J. H., Park E. S., Byun J. H. (2015). Gastric metastasis from primary lung adenocarcinoma mimicking primary gastric cancer. *World Journal of Gastrointestinal Oncology*.

[5] Uskent N., Baloğlu H., Çakmakçı M., Saglam S., Koksal U. (2016). Breast cancer metastases to the stomach and colon mimicking primary gastrointestinal cancer: four cases and literature review. *Advances in Modern Oncology Research*.

[6] Villa Guzman J. C., Espinosa J., Cervera R., Delgado M., Paton R., Cordero Garcia J. M. (2017). Gastric and colon metastasis from breast cancer: case report, review of the literature, and possible underlying mechanisms. *Breast Cancer: Targets and Therapy*.

[7] Namikawa T., Hanazaki K. (2014). Clinicopathological features and treatment outcomes of metastatic tumors in the stomach. *Surgery Today*.

[8] Xu L., Liang S., Yan N. (2017). Metastatic gastric cancer from breast carcinoma: a report of 78 cases. *Oncology Letters*.

[9] Ciulla A., Castronovo G., Tomasello G. (2008). Gastric metastases originating from occult breast lobular carcinoma: diagnostic and therapeutic problems. *World Journal of Surgical Oncology*.

[10] Kidney D. D., Cohen A. J., Butler J. (1997). Abdominal metastases of infiltrating lobular breast carcinoma: CT and fluoroscopic imaging findings. *Abdominal Imaging*.

[11] Arrangoiz R., Papavasiliou P., Dushkin H., Farma J. M. (2011). Case report and literature review: metastatic lobular carcinoma of the breast an unusual presentation. *International Journal of Surgery Case Reports*.

[12] Wasif N., Maggard M. A., Ko C. Y., Giuliano A. E. (2010). Invasive lobular vs. ductal breast cancer: a stage-matched comparison of outcomes. *Annals of Surgical Oncology*.

[13] Pectasides D., Psyrri A., Pliarchopoulou K. (2009). Gastric metastases originating from breast cancer: report of 8 cases and review of the literature. *Anticancer Research*.

[14] Taal B. G., Peterse H., Boot H. (2000). Clinical presentation, endoscopic features, and treatment of gastric metastases from breast carcinoma. *Cancer*.

[15] Riihimaki M., Hemminki A., Sundquist K., Sundquist J., Hemminki K. (2016). Metastatic spread in patients with gastric cancer. *Oncotarget*.

[16] Su W. C., Tsai H. L., Wu C. C. (2018). Two rare cases of synchronous and metachronous colonic metastases in patients with advanced gastric cancer. *World Journal of Surgical Oncology*.

[17] Gao B., Xue X., Tai W. (2014). Polypoid colonic metastases from gastric stump carcinoma: a case report. *Oncology Letters*.

[18] Jang H. J., Lim H. K., Kim H. S. (2001). Intestinal metastases from gastric adenocarcinoma: helical CT findings. *Journal of Computer Assisted Tomography*.

[19] Niu F. Y., Zhou Q., Yang J. J. (2016). Distribution and prognosis of uncommon metastases from non-small cell lung cancer. *BMC Cancer*.

[20] Lee P. C., Lo C., Lin M. T., Liang J. T., Lin B. R. (2011). Role of surgical intervention in managing gastrointestinal metastases from lung cancer. *World Journal of Gastroenterology*.

[21] Kim S. Y., Ha H. K., Park S. W. (2009). Gastrointestinal metastasis from primary lung cancer: CT findings and clinicopathologic features. *AJR. American Journal of Roentgenology*.

[22] Taira N., Kawabata T., Gabe A. (2017). Analysis of gastrointestinal metastasis of primary lung cancer: clinical characteristics and prognosis. *Oncology Letters*.

[23] Berger A., Cellier C., Daniel C. (1999). Small bowel metastases from primary carcinoma of the lung: clinical findings and outcome. *The American Journal of Gastroenterology*.

[24] Garwood R. A., Sawyer M. D., Ledesma E. J., Foley E., Claridge J. A. (2005). A case and review of bowel perforation secondary to metastatic lung cancer. *The American Surgeon*.

[25] Okazaki R., Ohtani H., Takeda K. (2010). Gastric metastasis by primary lung adenocarcinoma. *World Journal of Gastrointestinal Oncology*.

[26] Huang Q., Su X., Bella A. E. (2015). Clinicopathological features and outcome of gastric metastases from primary lung cancer: a case report and systematic review. *Oncology Letters*.

[27] Yang C. J., Hwang J. J., Kang W. Y. (2006). Gastro-intestinal metastasis of primary lung carcinoma: clinical presentations and outcome. *Lung Cancer*.

[28] Lee H. C., Yang M. T., Lin K. Y., Tu H. Y., Zhang T. A., Chen P. H. (2004). Metastases from gastric carcinoma to colon in the form of multiple flat elevated lesions: a case report. *The Kaohsiung Journal of Medical Sciences*.

[29] Dassen A. E., Lips D. J., Hoekstra C. J., Pruijt J. F., Bosscha K. (2009). FDG-PET has no definite role in preoperative imaging in gastric cancer. *European Journal of Surgical Oncology*.

[30] Arpino G., Bardou V. J., Clark G. M., Elledge R. M. (2004). Infiltrating lobular carcinoma of the breast: tumor characteristics and clinical outcome. *Breast Cancer Research*.

[31] Gadde R., Tamariz L., Hanna M. (2015). Metastatic gastric cancer (MGC) patients: can we improve survival by metastasectomy? A systematic review and meta-analysis. *Journal of Surgical Oncology*.

[32] Sarkut P., Ozer A., Gulcu B., Ozturk E., Gokgoz S., Ugras N. (2014). An extremely rare cause of gastric outlet: breast lobular carcinoma metastases to stomach. *The Breast Journal*.

[33] Oda I., Kondo H., Yamao T. (2001). Metastatic tumors to the stomach: analysis of 54 patients diagnosed at endoscopy and 347 autopsy cases. *Endoscopy*.

